# Increased incidence of acute kidney injury requiring dialysis in metropolitan France

**DOI:** 10.1371/journal.pone.0211541

**Published:** 2019-02-07

**Authors:** Fanny Garnier, Cécile Couchoud, Paul Landais, Olivier Moranne

**Affiliations:** 1 Nephrology-Dialysis-Apheresis Unit, Nîmes University Hospital, Nimes, France; 2 UPRES EA2415, Laboratory of Biostatistics, Epidemiology, Clinical Research and Health Economics, University of Montpellier, Montpellier, France; 3 REIN Registry, Biomedecine Agency, Saint Denis La Plaine, France; University of Sao Paulo Medical School, BRAZIL

## Abstract

**Background:**

Acute kidney injury requiring dialysis (AKI-D) is associated with high mortality. Information about its epidemiology is nonetheless sparse in some countries. The objective of this study was to assess its epidemiology and prognosis in metropolitan France.

**Methods:**

Using the French hospital discharge database, the study focused on adults hospitalized in metropolitan France between 2009 and 2014 and diagnosed with AKI-D according to the codes of the French common classification of medical procedures. Crude and standardized incidence rates (SIR) by gender and age were calculated. We explored the changes in patients’ characteristics, modalities of renal replacement therapy (RRT), in-hospital care, and mortality, along with their determinants. Trends over time in the SIR for AKI-D, its principal diagnoses, and comorbidities were analyzed with joinpoint models.

**Results:**

Between 2009 and 2014, the AKI-D SIR increased from 475 (95% CI, 468 to 482) to 512 per million population (95% CI, 505 to 519). AKI-D was twice as high in men as women. Median age was 68 years. Over the study period, the AKI-D SIR steadily increased in all age groups, particularly in the elderly. The most common comorbidities were cardio-cerebrovascular diseases (64.8%), pulmonary disease (42.2%), CKD (33.8%), and diabetes (26.0%); all of these except CKD increased significantly over time. In 2009, heart failure (17.2%), sepsis (17.0%), AKI (13.0%), digestive diseases (10.7%), and shock (6.6%) were the most frequent principal diagnoses, with a significant increase in heart failure and digestive diseases. The proportion of patients with at least one ICU stay and continuous RRT increased from 80.3% to 83.9% and from 56.9% to 61.8% (p<0.001), respectively. In-hospital mortality was high but stable (47%) and higher in patients with an ICU stay.

**Conclusions:**

This is the first exhaustive study in metropolitan France of the SIR for AKI-D. It shows this SIR has increased significantly over 6 years, together with ICU care and continuous RRT. In-hospital mortality is high but stable.

## Introduction

Acute kidney injury (AKI) is a common and severe complication in hospitalized patients [1,2]. Due to its prognosis and frequency, AKI has been identified as a global public health issue [3]. Whatever its etiology, AKI requiring dialysis (AKI-D) is associated with higher morbidity and mortality [4–6]. To develop preventive strategies and slow the growth of AKI-D, the International Society of Nephrology has recommended developing a better understanding of the epidemiology of AKI-D and its associated risk factors. Its global epidemiology, however, remains insufficiently characterized in the absence of accurate reporting of this event in many countries. Some studies from medico-administrative databases report dissimilar incidence rates over the past two decades [7–9]. Hsu et al. estimates that the crude incidence rate (CIR) of AKI-D rose from 222 to 533 per million population (pmp) between 2000 and 2009 in the United States [7], although a slightly more recent US study reports no increase in either the AKI-D incidence after adjustment for age and sex or in comorbidities between 2006 and 2014 [10]. In England, Kolhe et al. report that the CIR for AKI-D jumped from 15.9 to 208.7 pmp between 1998 and 2013 [8]. These increased incidence rates may be attributable to the growth of either susceptibility risk factors, such as advanced age, chronic kidney disease (CKD), and diabetes, or of exposure risk factors, such as nephrotoxic drugs or agents, sepsis, critical illness, and major surgery, together with changes in clinical practice related to indications for renal replacement therapy (RRT) [7,8,11,12]. Despite growing interest in AKI-D, national descriptions and prognostic data of these factors remain insufficient to enable the improvement of prevention and enhancement of healthcare organization.

In this context, our objective was to assess the trends in AKI-D incidence in metropolitan France between 2009 and 2014 and their determinants, from information in the French national hospital discharge database. Moreover, we explored the trends in patient characteristics, principal diagnoses, RRT modalities, hospital care, and mortality.

## Material and methods

### Study population

All hospitalizations for AKI-D from 2009 to 2014 were extracted from the comprehensive French hospital discharge database, which provides de-identified data with demographic and medical information about the admission diagnosis, underlying comorbidities, procedures performed, and complications. AKI-D was defined by three specific medical dialysis procedures and coded according to the French Common Classification of Medical Procedures (CCMP) [13] with the following codes for RRT: JVJF002 (RRT by intermittent hemodialysis, hemofiltration, or hemodiafiltration for AKI), JVJF005 (RRT by continuous hemodialysis, hemofiltration, or hemodiafiltration for AKI) and JVJB002 (RRT by peritoneal dialysis for AKI).

Patients aged younger than 18 years or not residents of metropolitan France (i.e., France excluding overseas districts and territories) were not included, nor were patients with a diagnosis of ESRD in the database before the AKI-D hospitalization. ESRD was defined by the codes presented in the supplementary data section. To reinforce the exclusion of ESRD patients, we applied a 3-month “wash-out” period and used the data for January-March, 2009, only to ensure that no patients from April had an ESRD diagnosis in the previous 3 months. Hospital stays for AKI-D in 2009 were estimated from the stays observed for the 9-month period that began in April after observing the absence of seasonal changes of AKI-D for years 2010–2014. To avoid bias from patients with recurrent AKI-D, new AKI-D episodes were retained only if they occurred at least six months after a previous episode, six months during which the patient had no stays for ESRD treated by dialysis.

### Data

The Agence Technique de l'Information Sur l'Hospitalisation (ATIH) waived the need for consent (decree No. 94–666). All French hospitals caring for medical and surgical patients submit de-identified patient data to the French hospital discharge database annually. The linking of each discharge summary submitted to this database to a national grouping algorithm leading to a French diagnosis-related group (DRG) [14] allowed patient comorbidities to be recorded and linked [15]. The study was conducted according to the approval given by the ATIH. Authorization was also obtained from the Commission Nationale de l'Informatique et des Libertés (agreement No. 1375062).

Hospitalization information included age and gender, admission from home or transfer from another hospital, admission to intensive care unit (ICU), modality of RRT (continuous or intermittent or peritoneal dialysis), interval from hospital admission to RRT initiation, length of stay, and in-hospital mortality. The principal diagnosis was the admission diagnosis: the condition for which the patient was admitted. Eight principal diagnosis groups were defined according to the ICD-10 (i.e., shock, respiratory disease, digestive disease, cardiac failure, sepsis, AKI, CKD, and other diagnoses). Associated comorbidities were defined according to ICD-10 codes [16]. Ten groups of comorbidities were defined according to the methodology of the French national health insurance fund (i.e., cardio-cerebrovascular, pulmonary, hepatic, and psychiatric comorbidities, CKD, diabetes mellitus, malignancy, obesity, malnutrition, and dementia)[17]. Cardio-cerebrovascular comorbidity included the following subgroups: rhythm disorders, heart failure, ischemic heart disease, peripheral vascular disease, stroke, and myocardial infarction. Because RRT modality could change during the course of hospitalization, three modalities were defined: continuous RRT (CRRT), intermittent RRT, and peritoneal dialysis (S1 Appendix).

#### Outcomes

Annual crude incidence of AKI-D was defined by the ratio of new AKI-D hospital admissions of adult residents of metropolitan France each year from 2009 to 2014. The annual AKI-D SIR was standardized for age and sex by direct standardization, with the adult French population in 2014 as reference. Incidence rates were expressed per million population per year.

### Statistical analysis

The characteristics of in-patients were expressed as absolute numbers and percentages according to calendar years. Qualitative values were compared with the chi-square test. Quantitative variables were described by their medians and interquartile ranges for non-Gaussian distributions and compared with the Wilcoxon score test and the Kruskal-Wallis test. The 95% confidence intervals of standardized incidence rates were calculated with a Poisson approximation.

Trends in standardized incidence rates of AKI-D, principal diagnoses, and comorbidities over the study period were analyzed with joinpoint models. A grid search was used to determine locations of no more than one joinpoint with a subsequent permutation test to determine superiority [18]. If the linearity of the trend was validated, the annual change was reported as the annual percent change (APC).

Patient characteristics and care were compared according to the outcome, either an ICU stay or in-hospital mortality. Univariate and multivariable logistic regression analyses were performed. The multivariable analysis used nested models containing several groups of variables, specifically, demographic data, year of admission, interval from admission to RRT initiation, comorbidities, principal diagnosis, and ICU admission, when applicable. Statistical analyses were performed by using SAS software version 9.3 (SAS Institute Inc.) and Joinpoint Statistical Software version 4.5.0.1 (Statistical Research and Applications Branch, National Cancer Institute).

## Results

### Population

**Fig 1:**
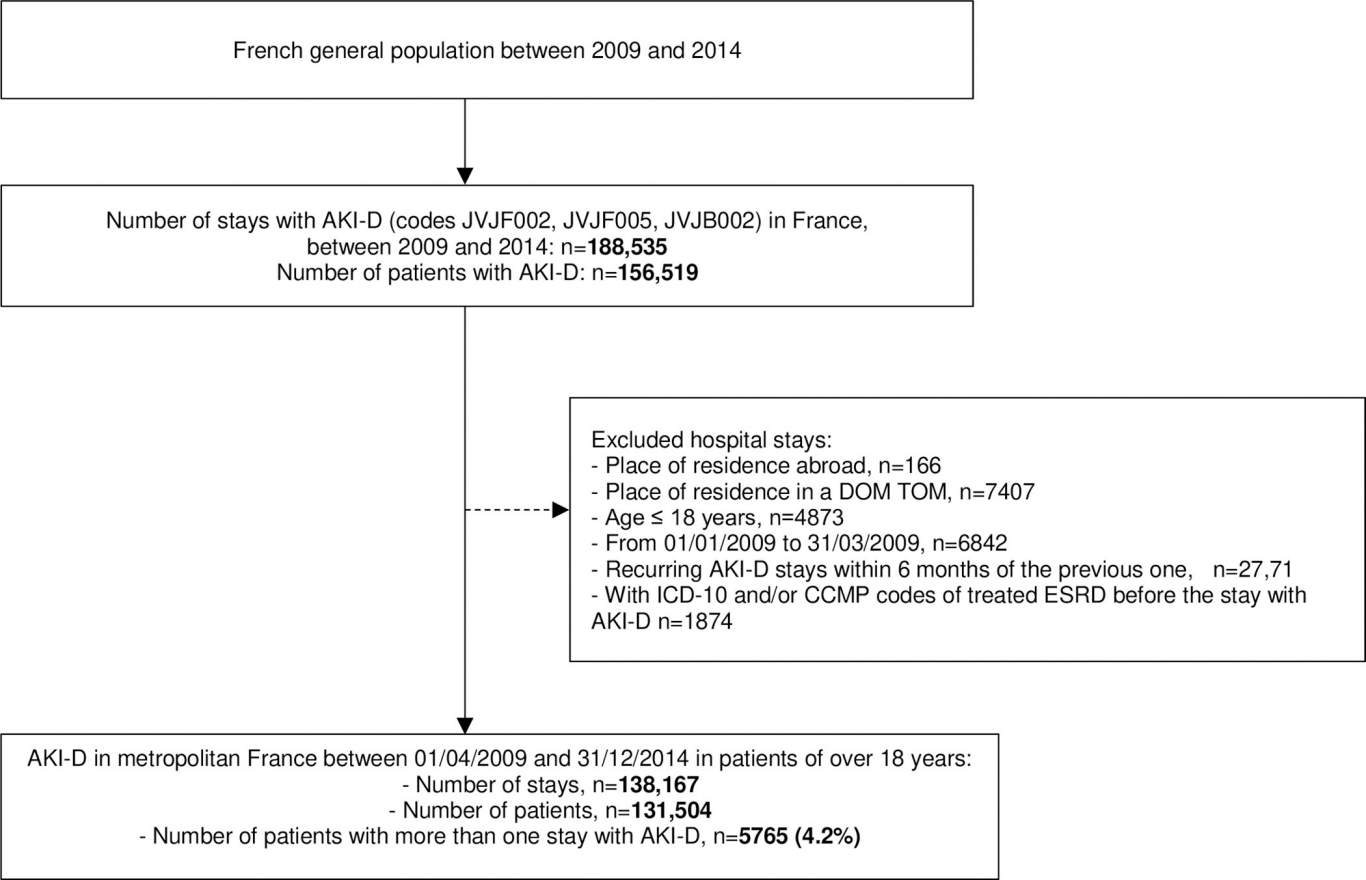
Flow chart. AKI-D, acute kidney injury requiring dialysis; ICD-10, International Classification of disease 10^th^ version; CCMP, Common Classification of Medical Procedures. DOM TOM, French overseas departments and territories.

Between April 1, 2009, and December 31, 2014, 122 million hospital stays were recorded in metropolitan France, for an estimated population of 64 million inhabitants. These included 138,167 hospital stays coded AKI-D of patients aged 18 years or more (Fig 1).

### Incidence trends of acute kidney injury requiring dialysis

**Fig 2:**
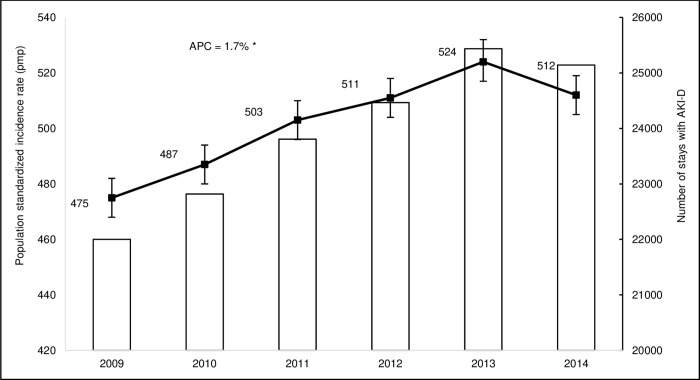
Population incidence of acute kidney injury requiring dialysis in metropolitan France from 2009 to 2014 (absolute number of stays and standardized incidence rate per million population). For 2009, the absolute number of stays and the crude incidence rate were reported as an estimation over 12 months based on observations for 9 months. I bars represent 95% CIs for standardized incidence rates. * indicates that the Annual Percent Change (APC) is significantly different from zero at the alpha = 0.05 level.

Between 2009 and 2014, the absolute number of stays with AKI-D increased from 22,001 to 25,142. The AKI-D SIR increased significantly from 475 (95% CI, 468 to 482) to 512 pmp (95% CI, 505 to 519) during this period with an APC of +1.7% (Fig 2).

### Trends in patient characteristics for stays with AKI-D

**Fig 3:**
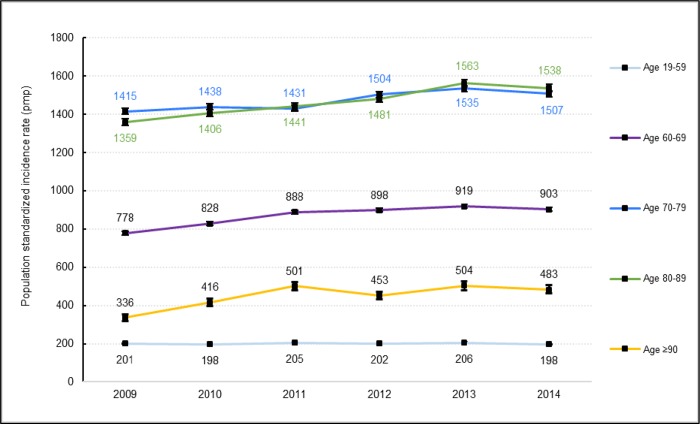
Population standardized incidence rate of acute kidney injury requiring dialysis in metropolitan France by age group from 2009 to 2014. For 2009, the standardized incidence rate was reported as an estimation over 12 months from observations over 9 months. I bars represent 95% CIs for standardized incidence rates.

**Fig 4:**
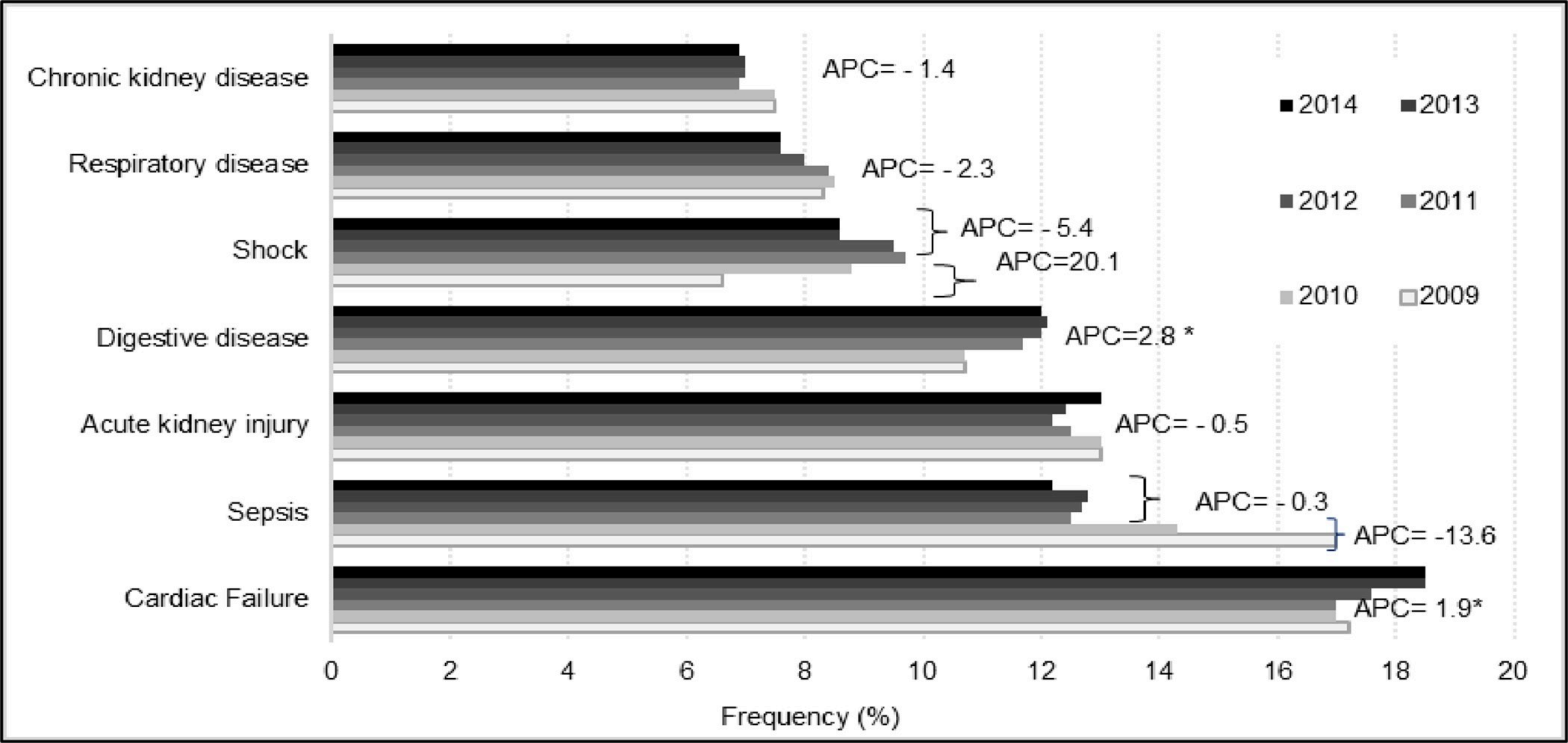
Trends in principal diagnoses of patients with AKI requiring dialysis from 2009 to 2014. * indicates that the Annual Percent Change (APC) is significantly different from zero at the alpha = 0.05 level.

Patients’ demographic and clinical characteristics are shown in Table 1 for the study period. The median age of population in 2009 was 68 years (interquartile range, 58–78); it was significantly higher by 2014. The APC of +0.36%. The SIR for AKI-D increased significantly in patients aged 60 years and older with increases of 16% for those 60–69 years, 7% for 70–79 years, 12% for 80–89 years, and 44% for those 90 years and older (Fig 3). The SIR for AKI-D by age and sex are shown in the supplementary data (S1 Fig). The most common comorbidities in 2009 were cardio-cerebrovascular (64.8%), pulmonary (42.2%), CKD (33.8%), diabetes (26.0%), and cancer (20.4%). Malnutrition, obesity, diabetes, and cardio-cerebrovascular and hepatic comorbidities increased significantly between 2009 and 2014 with APC of 12.3%, 5.6%, 2.9%, 1.4%, and 0.8%, respectively (S2 Fig). The six most commonly reported principal diagnoses during hospital stays in 2009 were heart failure (17.2%), sepsis (17.0%), AKI (13.0%), digestive diseases (10.7%), respiratory diseases (8.3%), and shock (6.6%), with significant and linear increases subsequently observed for heart failure (APC 2.8%) and digestive diseases (APC 1.9%) (Fig 4).

**Table 1:** Trends in demographic and clinical characteristics of AKI patients requiring dialysis in metropolitan France from 2009 to 2014.

	2009	2010	2011	2012	2013	2014	p-value*
Number of stays with AKI-D (%)	16 500 (11.9)	22 818 (16.5)	23 806 (17.2)	24 465 (17.7)	25 436 (17.2)	25 142 (18.2)	<0.001
Characteristics							
Men (%)	10 606 (64.3)	14 752 (64.6)	15 552 (65.3)	15 934 (65.1)	16 534 (65.0)	16 282 (64.8)	0.28
Median age (IQR), yr	68.0[57–77]	68.0[57–77]	68.0[57–77]	68.0[58–78]	68.0[58–78]	68.0[59–78]	<0.001
Age group (%)							
19–59 years	5118 (31.0)	6714 (29.4)	6915 (29.0)	6813 (27.8)	6911 (27.2)	6642 (26.4)	<0.001
60–69 years	3560 (21.6)	5268 (23.1)	5915 (24.8)	6240 (25.5)	6615 (26.0)	6678 (26.6)	
70–79 years	4913 (29.8)	6653 (29.2)	6544 (27.5)	6783 (27.7)	6900 (27.1)	6825 (27.1)	
80–89 years	2808 (17.0)	4012 (17.6)	4187 (17.6)	4376 (17.9)	4702 (18.5)	4678 (18.6)	
≥90 years	101 (0.6)	171 (0.7)	245 (1.0)	253 (1.0)	308 (1.2)	319 (1.3)	
Comorbidities (%)							
Cardio-cerebrovascular comorbidities	10 687 (64.8)	14 903 (65.3)	15 855 (66.6)	16 652 (68.1)	17 532 (68.9)	17 352 (69.0)	<0.001
*Rhythm disorder*	4548 (27.6)	6556 (28.7)	7187 (30.2)	7820 (32.0)	8365 (32.9)	8188 (32.6)	<0.001
*Heart failure*	4184 (25.4)	5524 (24.2)	5625 (23.6)	6119 (25.0)	6452 (25.4)	6289 (25.0)	<0.001
*Ischemic heart disease*	3780 (22.9)	4993 (21.9)	5336 (22.4)	5616 (23.0)	5812 (22.9)	5581 (22.2)	0.03
*Peripheral vascular disease*	1369 (8.3)	2003 (8.8)	2165 (9.1)	2295 (9.4)	2209 (8.7)	2216 (8.8)	0.004
*Stroke*	866 (5.2)	1173 (5.1)	1339 (5.6)	1313 (5.4)	1404 (5.5)	1393 (5.5)	0.17
*Myocardial infarction*	298 (1.8)	472 (2.1)	473 (2.0)	502 (2.1)	538 (2.1)	536 (2.1)	0.24
Pulmonary comorbidities	6965 (42.2)	9107 (39.9)	9788 (41.1)	10478 (42.8)	10916 (42.9)	10771 (42.8)	<0.001
Chronic kidney disease	5580 (33.8)	7255 (31.8)	7637 (32.1)	7844 (32.1)	8240 (32.4)	8296 (33.0)	<0.001
Diabetes mellitus	4294 (26.0)	6165 (27.0)	6298 (24.5)	6868 (28.1)	7461 (29.3)	7514 (29.9)	<0.001
Malignancy	3372 (20.4)	4474 (19.6)	4761 (20.0)	4877 (19.9)	5074 (19.9)	5119 (20.4)	0.29
Psychiatric comorbidities	2436 (14.8)	3638 (15.9)	3929 (16.5)	4160 (17.0)	4194 (16.5)	4325 (17.2)	<0.001
Liver comorbidities	2322 (14.1)	3266 (14.3)	3397 (14.3)	3634 (14.8)	3741 (14.7)	3662 (14.6)	0.18
Obesity	1783 (10.8)	2596 (11.4)	2853 (12.0)	3265 (13.3)	3431 (13.5)	3511 (14.0)	<0.001
Malnutrition	692 (4.2)	1069 (4.6)	1277 (5.4)	1559 (6.4)	1717 (6.7)	1830 (6.3)	<0.001
Dementia	205 (1.2)	307 (1.3)	284 (1.2)	316 (1.3)	318 (1.2)	344 (1.4)	0.53
Principal diagnosis (%)							
Heart failure	2833 (17.2)	3889 (17.0)	4056 (17.0)	4298 (17.6)	4708 (18.5)	4641 (18.5)	<0.001
Sepsis	2808 (17.0)	3254 (14.3)	2972 (12.5)	3102 (12.7)	3244 (12.8)	3064 (12.2)	<0.001
Acute kidney injury	2147 (13.0)	2966 (13.0)	2978 (12.5)	2989 (12.2)	3165 (12.4)	3271 (13.0)	0.02
Digestive disease	1769 (10.7)	2442 (10.7)	2791 (11.7)	2931 (12.0)	3082 (12.1)	3021 (12.0)	<0.001
Shock	1089 (6.6)	2005 (8.8)	2297 (9.7)	2312 (9.5)	2193 (8.6)	2151 (8.6)	<0.001
Pulmonary disease	1372 (8.3)	1937 (8.5)	2003 (8.4)	1961 (8.0)	1925 (7.6)	1898 (7.6)	<0.001
Chronic kidney disease	1214 (7.4)	1712 (7.5)	1647 (6.9)	1702 (7.0)	1795 (7.1)	1726 (6.9)	0.04
Other diagnosis	3269 (19.9)	4613 (20.5)	5062 (19.1)	5170 (20.9)	5324 (20.9)	5370 (21.6)	<0.001

Abbreviation: AKI-D, acute kidney injury requiring dialysis; IQR, interquartile range.

For 2009, the absolute number of stays and clinical characteristics were estimated from observations over 9 months.

*The p value denotes a global difference between 2009 and 2014 according to the Chi-Square test.

### Patient trajectory and care

Most patients had at least one ICU admission during their hospital stay, with a significant increase from 80.3% to 83.9% over time. Globally, the use of CRRT (at least one use) rose significantly from 56.9% in 2009 to 61.8% in 2014 (Table 2), primarily in ICU, where use increased significantly from 67.9% to 71.3% (Table 3).

**Table 2:** Changing patterns in management of stays with AKI requiring dialysis between 2009 and 2014 in metropolitan France.

	2009	2010	2011	2012	2013	2014	p-value*
Provenance							
Home (%)	13 995 (84.8)	19 377 (84.2)	20 205 (84.9)	20 700 (84.6)	21 469 (84.4)	21 236 (84.5)	0.49
Median delay to starting dialysis (IQR), days	1[0–4]	1[0–6]	1[0–6]	1[0–6]	2[0–6]	2[0–6]	<0.001
Type of RRT (%)							
At least once continuous RRT	9398 (56.9)	13 089 (57.4)	13 997 (58.8)	14 730 (60.2)	15 605 (61.4)	15 527 (61.8)	<0.001
Intermittent RRT alone	7034 (42.6)	9643 (42.3)	9711 (40.8)	9629 (39.4)	9711 (38.2)	9541 (37.9)	<0.001
Peritoneal dialysis alone	69 (0.4)	86 (0.4)	98 (0.4)	106 (0.4)	120 (0.5)	74 (0.3)	0.04
Admission to ICU (%)	13 250 (80.3)	18 516 (81.5)	19 466 (81.8)	20 169 (82.4)	21 273 (83.6)	21 089 (83.9)	<0.001
Median length of stay (IQR), days	17 [7–34]	17 [7–34]	17 [7–34]	17 [7–34]	17[6–33]	17[7–34]	0.002

Abbreviation: ICU, intensive care unit; IQR, interquartile range; RRT, renal replacement therapy.

For 2009, data were estimated from observations over 9 months.

* The p value denotes a global difference between 2009 and 2014 according to the Chi-Square test.

**Table 3:** Changing patterns in patient characteristics and management of stays with AKI requiring dialysis in intensive care units between 2009 and 2014 in metropolitan France.

	2009	2010	2011	2012	2013	2014	p-value*
Number of stays (%)	13 250 (80.3)	18 516 (81.5)	19 466 (81.8)	20 169 (82.4)	21 273 (83.6)	21 089 (83.9)	<0.001
Characteristics							
Men (%)	8608 (65.0)	12 084 (65.2)	12 891 (66.2)	13 279 (65.8)	13 906 (65.4)	13 815 (65.5)	0.2
Median age (IQR), yr	68 [56–77]	67 [57–77]	67 [57–77]	67 [58–77]	68 [58–77]	68 [58–77]	<0.001
Median IGS2 (IQR)	59 [45–75]	59 [45–75]	59 [46–76]	60 [46–77]	60 [46–77]	60 [46–77]	<0.001
Mechanical ventilation (%)	10 319 (77.9)	14 504 (78.3)	15 203 (78.1)	15 739 (78.0)	16 475 (77.4)	16 050 (76.1)	<0.001
Vasopressors (%)	9855 (74.4)	13 962 (75.4)	14 741 (75.7)	15 296 (75.8)	16 153 (75.9)	15 977 (75.6)	0.02
Type of RRT (%)							
At least one continuous RRT	8984 (67.9)	12 622 (68.3)	13 589 (69.9)	14 271 (70.9)	15 130 (71.3)	15 003 (71.3)	<0.001
Intermittent RRT, alone	4244 (32.1)	5862 (31.7)	5847 (30.1)	5869 (29.1)	6091 (28.7)	6055 (28.7)	<0.001
Admission-RRT interval (%)							
0–1 day	7871 (59.4)	9457 (51.1)	9886 (50.8)	9900 (49.1)	10 391 (48.8)	10 231 (48.5)	
2–7 days	2889 (21.8)	4847 (26.2)	5245 (26.9)	5639 (28.0)	5949 (28.0)	6057 (28.7)	<0.001
≥8 days	2490 (18.8)	4212 (22.7)	4335 (22.3)	4630 (23.0)	4933 (23.2)	4801 (22.8)	

Abbreviation: IQR, interquartile range; IGS2, Index of Gravity score 2; RRT, renal replacement therapy.

* The p value denotes a global difference between 2009 and 2014 according to the Chi-Square test.

For 2009, the absolute number of stays and others characteristics were estimated from observations over 9 months.

### Characteristics of the population according to ICU admission

Characteristics of the population according to ICU admission are shown in the supplementary data S1 Table and S3 Fig. In the multivariable analysis, younger age, later year of admission during the study period, principal diagnosis, and comorbidities (*i*.*e*., hepatic, pulmonary, and cardio-cerebrovascular comorbidities and cancer) were associated with a risk of ICU admission (S2 Table).

### In-hospital mortality rate, trends, and risk factors

In-hospital mortality remained stable at 46.6% during the study period, significantly higher in men (47.4%) than in women (45.1%) (p<0.001). In the multivariable analysis, gender, age, interval between admission and RRT initiation, comorbidities, principal diagnosis, and ICU admission were significantly associated with in-hospital mortality (Table 4). Among patients with at least one ICU admission, mortality decreased significantly from the start to the end of the study period, from 54.3% to 51.7% (S4 Fig).

**Table 4:** Univariate and multivariable analyses of in-hospital mortality in AKI patients requiring dialysis.

	Number of death, n(%)	Univariate analysis OR [95% CI]	Multivariable ^a^ OR [I95% CI]	Multivariable ^b^ OR [95% CI]
Gender				
Men	42 474 (47.4)	1 (Reference)	1 (Reference)	1 (Reference)
Women	21 856 (45.1)	**0.91[0.89–0.93]**	0.97[0.95–1.00]	0.93[0.91–0.95]
Age groups				
19–59 years	15 937 (39.3)	1 (Reference)	1 (Reference)	1 (Reference)
60–69 years	15 617 (45.6)	**1.29[1.25–1.33]**	**1.44[1.40–1.49]**	**1.32[1.28–1.37]**
70–79 years	19 511 (50.5)	**1.57 [1.53–1.62]**	**2.05[1.99–2.12]**	**1.73[1.68–1.79]**
80–89 years	13 130 (53.2)	**1.74 [1.68–1.79]**	**2.87[2.77–2.99]**	**2.42[2.33–2.51]**
≥90 years	675 (48.3)	**1.44[1.29–1.60]**	**3.74[3.31–4.23]**	**3.29[2.90–3.72]**
Years				
2009	7680 (46.5)	1 (Reference)	1 (Reference)	1 (Reference)
2010	10 681 (46.8)	1.01[0.97–1.05]	0.96[0.92–1.01]	0.97[0.93–1.01]
2011	11 068 (46.5)	0.99[0.96–1.04]	**0.94[0.90–0.98]**	**0.93[0.89–0.98]**
2012	11 528 (47.1)	1.02[0.98–1.06]	**0.94[0.90–0.98]**	**0.94[0.90–0.99]**
2013	11 826 (46.5)	0.99[0.96–1.04]	**0.90[0.86–0.94]**	**0.91[0.87–0.95]**
2014	11 547 (45.9)	0.97[0.94–1.01]	**0.88[0.84–0.92]**	**0.88[0.85–0.92]**
Admission-RRT interval				
0–1 day	30 020 (42.0)	1 (Reference)	1 (Reference)	1 (Reference)
2-7days	17 717 (47.4)	**1.24[1.21–1.27]**	**1.18[1.14–1.21]**	**1.13[1.01–1.16]**
>8 days	16 593 (56.7)	**1.81[1.76–1.86]**	**1.58[1.53–1.63]**	**1.52[1.47–1.56]**
Comorbidities				
Cardio-cerebrovascular comorbidities	18 993 (42.0)	**1.55[1.51–1.59]**	**1.17[1.14–1.20]**	—
Diabetes mellitus	15 196 (39.4)	**0.79[0.77–0.81]**	**0.74[0.72–0.76]**	—
Pulmonary comorbidities	33 145 (57.1)	**1.90[1.85–1.94]**	**1.51[1.48–1.55]**	—
Malignancy	15 082 (54.5)	**1.45[1.41–1.49]**	**1.34[1.30–1.38]**	—
Liver comorbidities	12 141 (60.6)	**1.85[1.80–1.91]**	**1.95[1.89–2.02]**	—
Obesity	7241 (41.5)	**0.78[0.76–0.81]**	**0.78[0.75–0.81]**	—
Chronic kidney disease	12 724 (28.4)	**0.35[0.34–0.36]**	**0.49[0.39–0.41]**	—
Principal diagnosis				
Shock	7748 (64.3)	**20.54[18.93–22.29]**	—	**9.47[8.69–10.33]**
Respiratory disease	6925 (62.4)	**18.92[17.43–20.55]**	—	**8.06[7.39–8.78]**
Digestive disease	9870 (61.6)	**18.24[16.85–19.75]**	—	**7.99[7.35–8.69]**
Sepsis	10 560 (57.2)	**15.27[14.12–16.51]**	—	**6.98[6.43–7.59]**
Heart failure	11 171 (45.7)	**9.61[8.89–10.37]**	—	**4.39[4.05–4.76]**
Other diagnosis	12 821 (44.5)	**9.14[8.45–9.87]**	—	**4.86[4.48–5.26]**
Acute kidney injury	4445 (25.4)	**3.88[3.58–4.20]**		**2.22[2.04–2.41]**
Chronic kidney disease	790 (8.1)	1 (Reference)		1 (Reference)
Admission to ICU				
0	3684 (15.1)	1 (Reference)	1 (Reference)	1 (Reference)
1	60 646 (53.3)	**6.42[6.19–6.66]**	**4.73[4.55–4.93]**	**4.31[4.14–4.48]**

Abbreviation: OR, Odds ratio; ICU, intensive care unit; RRT, renal replacement therapy.

^a^ Adjusted for gender, age group, year, admission-RRT interval, ICU admission, and comorbidities.

b Adjusted for gender, age group, year, admission-RRT interval, ICU admission, and comorbidities and principal diagnosis.

Odds ratios and confidence intervals in bold indicate statistical significance.

### Patient characteristics and changes in management in patients with an ICU admission

For patients with at least one admission to ICU, the median IGS2 was statistically significantly higher at the end of the study period, rising from 59 (IQR, 45–75) to 60 (IQR, 46–77). The difference in the frequency of mechanical ventilation was also statistically significant, dropping from 77.9% to 76.1%. Conversely, the use of vasopressors was significantly higher at the end of the study period, as were the use of the CRRT modality and interval between admission and RRT initiation (Table 3).

## Discussion

For this first study of AKI-D in metropolitan France, using the national hospital discharge database, we showed a significant increase in the AKI-D SIR in adults from 475 to 512 pmp over the study period (2009–2014). This corresponds to an APC of +1.7%. In 2014, the incidence of AKI-D was more than three times higher than that of ESRD (163 pmp) [19]. Note that this result is adjusted for population age and sex. We also reported an increase in several comorbidities, notably cardio-cerebrovascular, and described the modalities of dialysis used to manage AKI. As commonly reported, the principal diagnoses for these hospital stays were diverse: AKI, heart failure, sepsis, digestive diseases, respiratory diseases, and shock, with a significant increase in heart failure and digestive diseases. In 2014, most patients experienced at least one admission to ICU (83.9%) and at least one treatment by CRRT (61.8%); both of these rates increased significantly during the study period. Overall in-hospital mortality was higher in ICU than in non-ICU patients, but it decreased significantly from 54.3% to 51.7% over the study period.

### Comparisons with other studies

Our results are consistent with the results of previous studies in the United States of America, England, and Denmark [7–9,20,21]. In the US, the crude incidence of AKI-D was higher than in Europe and increased from 222 to 533 pmp between 2000 and 2009 [7]. This US incidence of AKI-D might, nonetheless, be biased because it was calculated from a nationwide inpatient sample and with a weighting system. It might also be overestimated because patients with ICD-9 codes of ESRD were not excluded. Because the US results by Kashani et al. found no increase in AKI-D incidence after age and sex adjustments, our results differ from theirs [10]. Unlike them, we used as the denominator of our incidence rates the “at risk” population, i.e., the population of metropolitan France, rather than hospitalized patients. The incidence rate of AKI-D in our study was somewhat higher than that observed in England and Denmark for the same period [8,9]. This result might be due in part to the use of different indications for RRT in clinical practice.

Another point of difference is that our incidence rates were standardized. Others have showed an increase of crude incidence of AKI-D without standardization and without considering the aging of the general population [7–9]. Previous studies of ICU patients have reported that CRRT is the most widely-used RRT for AKI-D (between 52% to 80%) [9,22–24]. We showed an increased incidence of AKI-D related to at least one CRRT modality in ICU. In our study, in-hospital mortality was stable at 47% and was higher than that either in England (41.1% in 2008–2013) [8] or in the US national administrative database study reporting that in-hospital mortality decreased from 28% to 19.7% between 2001 and 2011 [25]. However, our hospital mortality was lower than that reported by Uchino et al. (60.3%) in their multinational study in 2001 of patients with ICU stays [24].

### Determinants of the increasing incidence rate of AKI-D

The rise in the SIR for AKI-D in metropolitan France might be due either to an increase in the population requiring acute RRT, or to a change in the indications for RRT in clinical practice, or both.

The first hypothesis finds support in the increase in severe forms of AKI requiring RRT, associated with an increasing susceptibility to AKI due to several risk factors, including aging, cardio-cerebrovascular and hepatic comorbidities, diabetes, and obesity [7–9,26,27]. Furthermore, heart failure is a well-known risk factor for AKI-D, especially for patients with vascular overload meeting the definition of the cardiorenal syndrome; this too could account for the increasing AKI-D incidence [7,28–30]. Another well-known AKI risk factor is sepsis [24,31–34], but its frequency with sepsis decreased in our study, presumably associated with better management of sepsis and/or changes in RRT indications in recent years. Hsu et al. showed that septicemia, hypertension, respiratory failure, coagulation disorders, shock, and liver disease account for the increase in the temporal trend of AKI-D. Several of these factors were present in our study [35]. Furthermore, the stability of CKD comorbidity over 30% during the study period suggests that the increase is not explained by AKI-D among CKD patients, but by severe AKI requiring dialysis in patients with previously normal kidney function. Moreover, the interval between admission and RRT initiation increased, corresponding to an increase in hospital-acquired AKI compared to admission for community-acquired AKI [36].

Our second hypothesis suggests a change in RRT indications in clinical practice, more specifically, in ICUs, which are using RRT more liberally. CRRT in routine clinical practice began only in the late 1990s, together with improvement in technical modalities and new indications over time, such as sepsis, for which early CRRT is now indicated [37–43]. Our results suggest that intensivists increasingly consider RRT to be indicated for patients with circulatory failure or with multiple comorbidities. This, combined with better availability of the continuous modalities, may account for the increased use of CRRT in the ICU [7,37,38,44–46]. In Denmark, increased incidence of AKI-D coincided with a growth in CRRT from 27.4% to 56.4% between 2000 and 2012; moreover, CRRT patients were all admitted to ICU [9]. The increase in AKI-D incidence could also be explained by an increase in the use of RRT for severe AKI in patients aged 70–89 years. In very elderly patients over 90 years, the incidence of AKI-D was much lower, despite its high rate of increase: because of their poor prognosis, these patients are rarely admitted to ICU and rarely undergo invasive procedures such as RRT when they are needed.

### Strengths and limitations of the study

Our study has several strengths. First, it was based on the French hospital discharge database, which records all public and private hospitalizations and is complete for RRT. Although it is retrospective, this report is based on prospective recording of all hospitalizations over the study period and thus minimizes information bias. Second, it provides an accurate estimation of the SIR for AKI-D applicable to metropolitan France as a whole. Third, coding of RRT for AKI has appeared to be a robust criterion in hospital discharge databases that has proven in previous studies and in other countries that use it for billing and reimbursement purposes [47,48] to be sensitive (76.9%-90.3%) and highly specific (93.8%-99.9%). Finally, our study was based on an algorithm that identified all AKI-D cases. This algorithm excluded patients with an ESRD code in any hospitalization before the AKI-D hospital stay and any recurrent AKI-D stays less than 6 months after an included stay for AKI-D. This limited the potential overestimation of the AKI-D incidence rate.

Our study must nonetheless be interpreted in light of the following limitations. First, neither the cause of AKI nor the indication for RRT appeared as such in the discharge summary, which did, however, include the reason for admission. A second limitation is the risk of misclassification between AKI-D and ESRD when both AKI-D and ESRD codes were present for the same stay, as they were in 24% of stays. In the study by Hsu et al., among the AKI-D hospitalizations, 36% had concurrent discharge diagnostic codes of ESRD [35]. In a sensitivity analysis excluding hospitalized stays that had both AKI-D and ESRD codes recorded, we found that both crude and standardized incidence rates of AKI-D showed a rising temporal trend during the study period (2009–2014). These hospital stays might correspond to ESRD patients starting RRT in emergency conditions, to AKI-D complicated with ESRD that indicated the need for post-discharge RRT, or to ESRD that was incorrectly coded. A study in California showed that many patients with AKI-D did not recover renal function and were considered to have reached ESRD [5]. Another limitation of our study is an information bias secondary to the quality of coding of comorbidities and principal diagnoses. Thus, in some cases, CRRT patients might have received intermittent dialysis during their hospital stay; however, only 20% of patients were treated with both modalities, and this rate decreased during the study period. Finally, we restricted the study to AKI-D patients because of missing information about KDIGO classification in the national hospital discharge database. However, it is essential to learn more about the epidemiology of AKI-D to develop preventive strategies for improving prognosis and decreasing costs.

### Clinical implications

We provide additional support of the rising incidence of AKI-D from 2009 to 2014 in metropolitan France, further confirming the need for steps to be taken upstream to decrease risk factors and prevent AKI. Moreover, the indication for RRT in AKI must be appraised, specifically when associated with other conditions such as cardiac failure and sepsis. Furthermore, health services need to be alerted so that they can adapt and enrich the provision of care. Finally, a prospective study would be useful to better understand the indications for RRT in ICUs and thus improve the RRT initiation strategy there [49,50].

## Supporting information

S1 Fig Standardized incidence rate of acute kidney injury requiring dialysis in metropolitan France, stratified by age and sex, from 2009 to 2014. (TIFF)

S2 Fig Trends in comorbidities of patients with AKI requiring dialysis from 2009 to 2014 in metropolitan France. (TIFF)

S3 Fig Comparison of the frequency of principal diagnoses according to place of care in metropolitan France. (TIFF)

S4 Fig Comparison of the frequency of death according to place of care in metropolitan France. (TIFF)

S1 Table Characteristics of population according to ICU admission during hospital stays in metropolitan France between 2009 and 2014. (TIFF)

S2 Table Univariate and multivariable analyses of admission to intensive care units of AKI patients requiring dialysis. (TIFF)

S1 Appendix (TIFF)
