# Supplementary figures and images for: Increased incidence of acute kidney injury requiring dialysis in metropolitan France
Source: PLoS One. 2019 Feb 7;14(2):e0211541. doi: 10.1371/journal.pone.0211541 (PMC6366739; doi:10.1371/journal.pone.0211541)

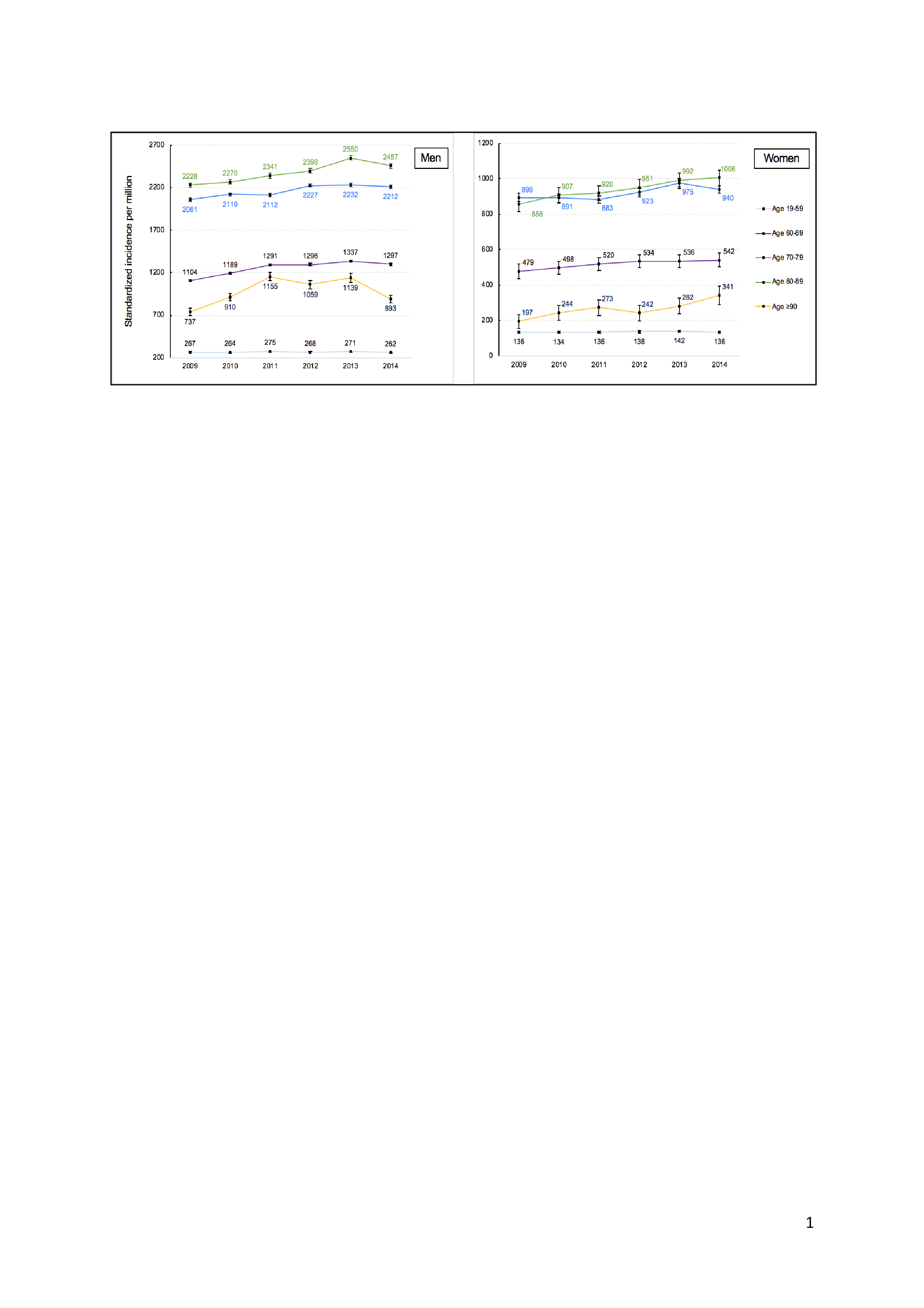

Supplement: S1 Fig — (TIFF) [file pone.0211541.s001.tiff]

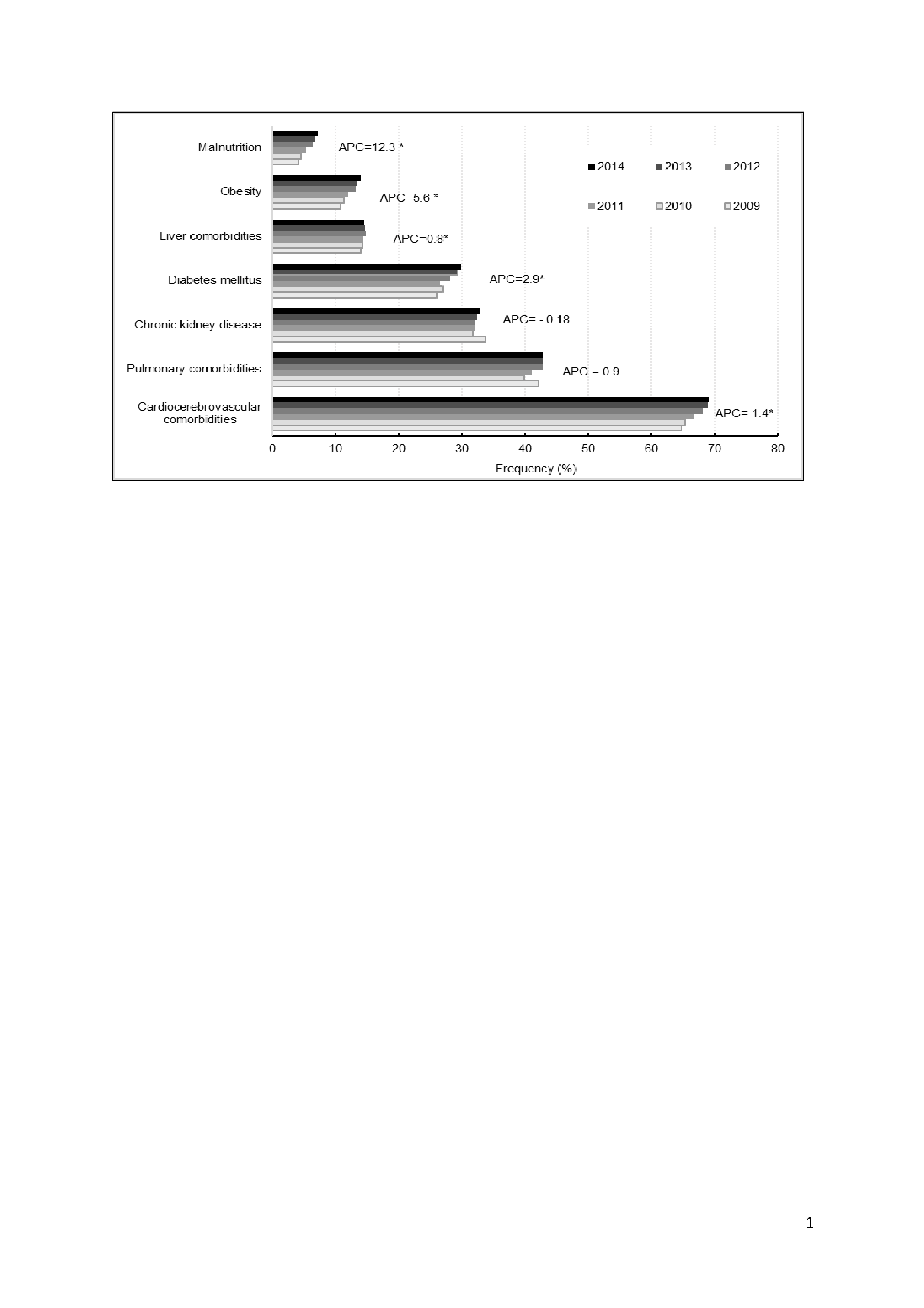

Supplement: S2 Fig — (TIFF) [file pone.0211541.s002.tiff]

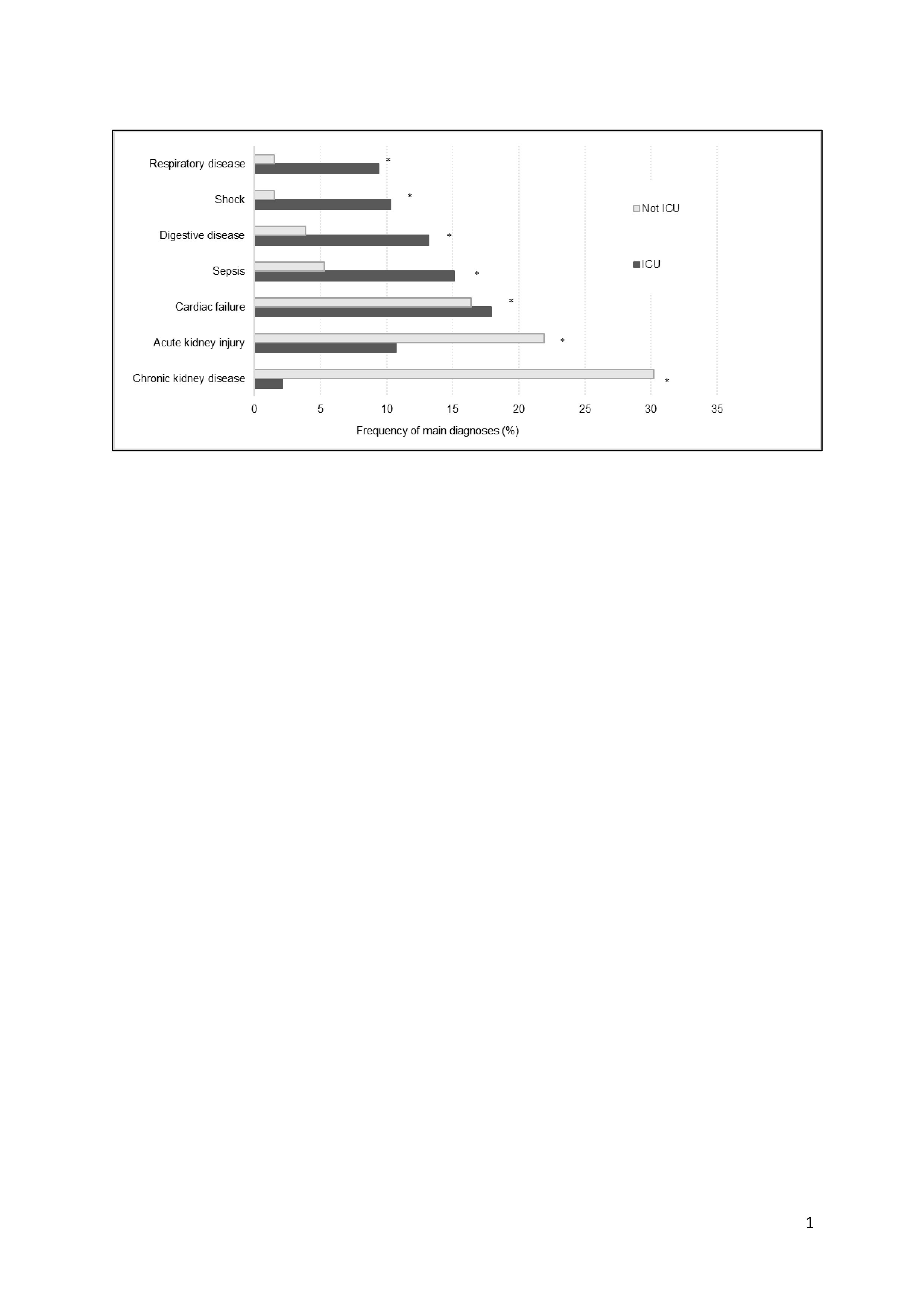

Supplement: S3 Fig — (TIFF) [file pone.0211541.s003.tiff]

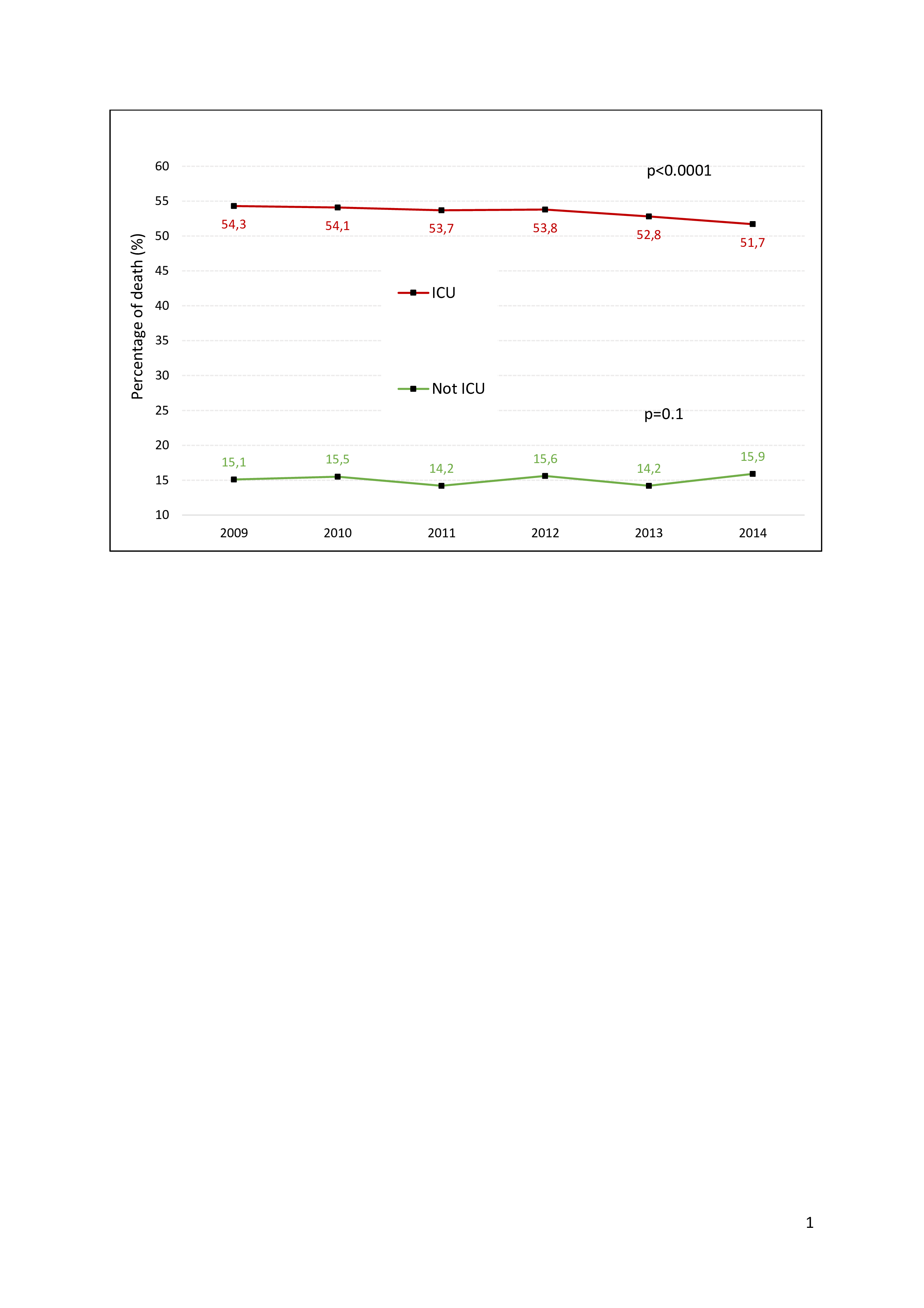

Supplement: S4 Fig — (TIFF) [file pone.0211541.s004.tiff]

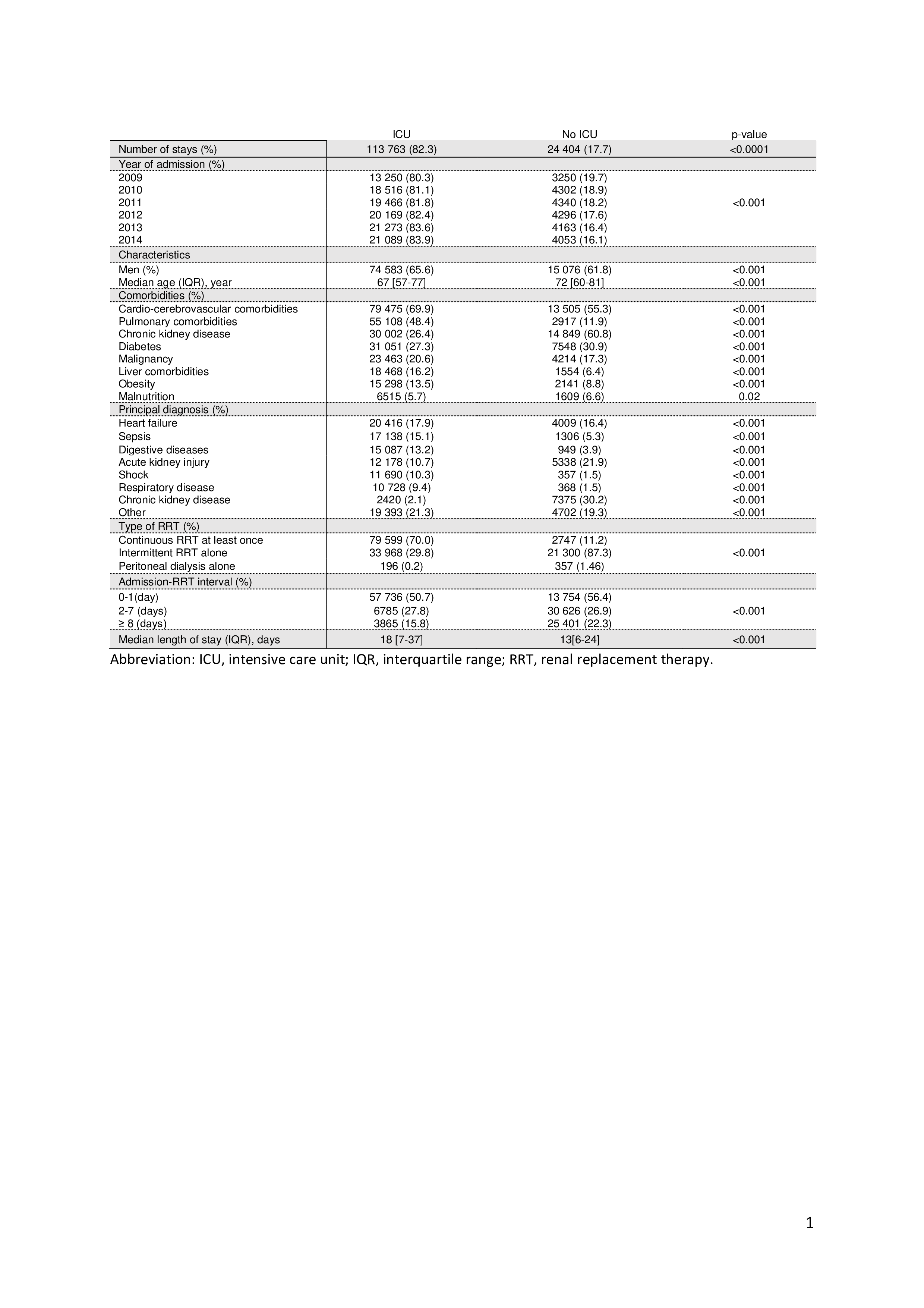

Supplement: S1 Table — (TIFF) [file pone.0211541.s005.tiff]

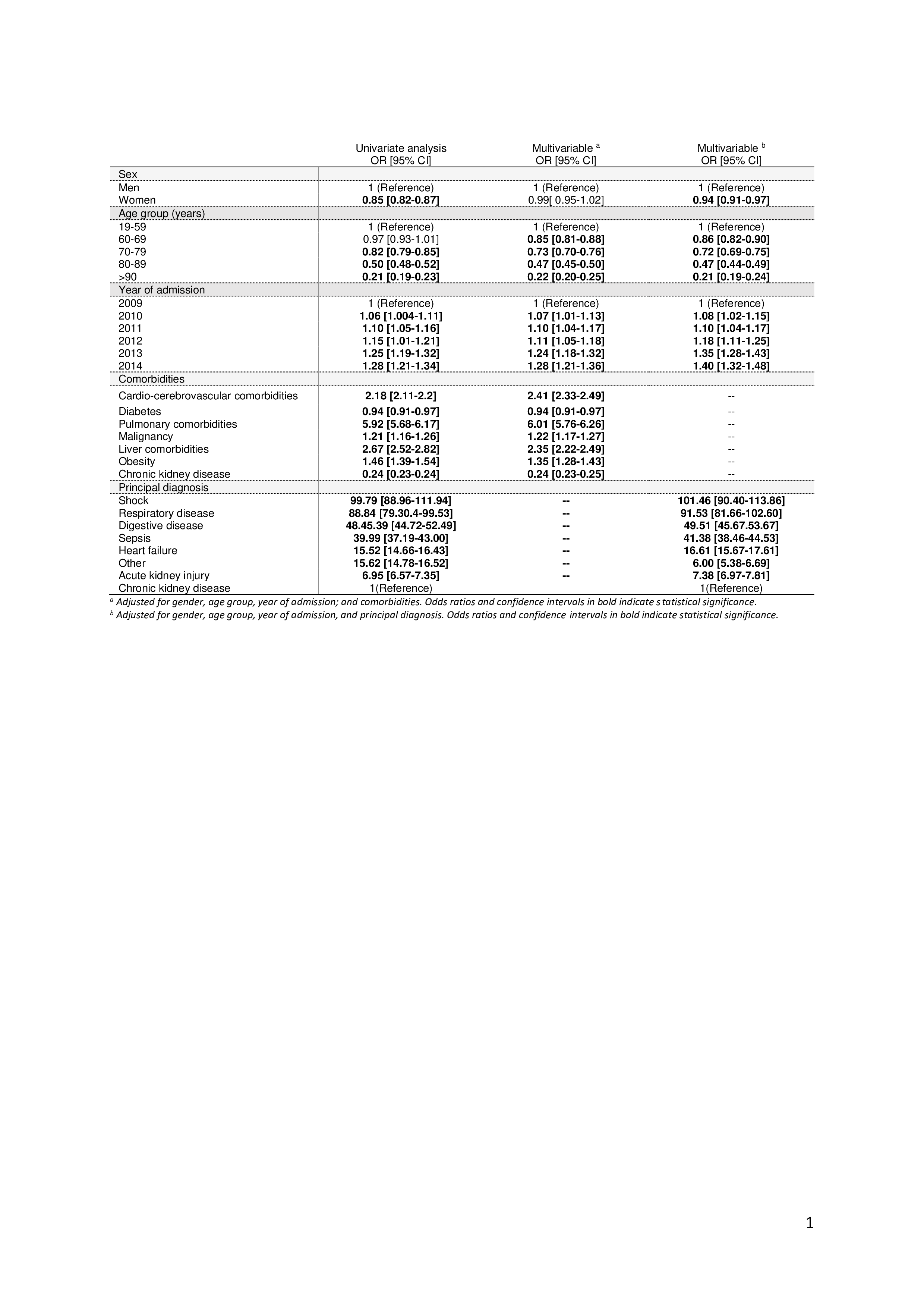

Supplement: S2 Table — (TIFF) [file pone.0211541.s006.tiff]

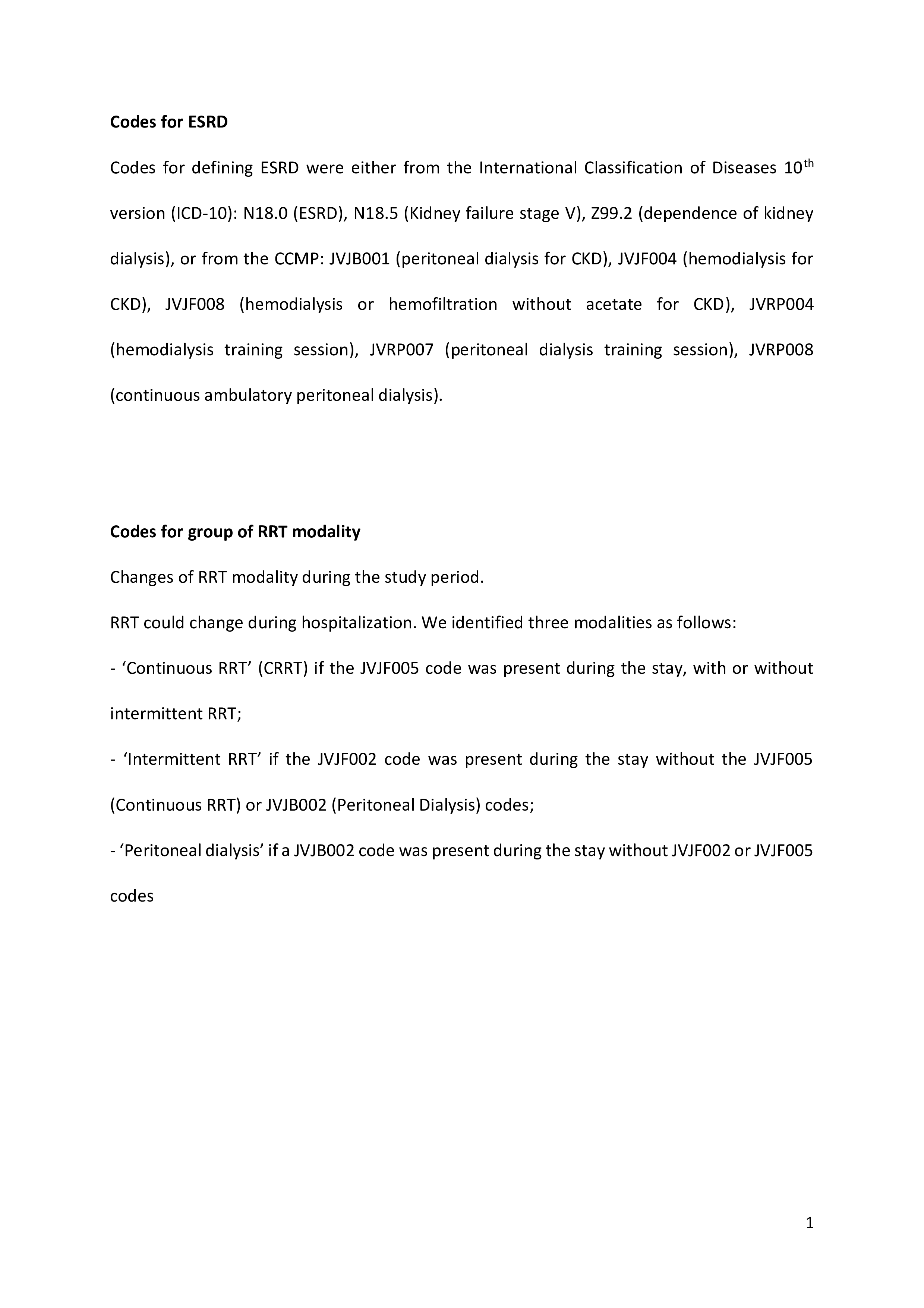

Supplement: S1 Appendix — (TIFF) [file pone.0211541.s007.tiff]
